# Academic Identity and Self-Regulation Strategies During the Transition to College: The Roles of Quiet Ego and Self-Esteem

**DOI:** 10.3390/bs16040489

**Published:** 2026-03-26

**Authors:** Heidi A. Wayment

**Affiliations:** Psychological Sciences, Northern Arizona University, Flagstaff, AZ 86011, USA; heidi.wayment@nau.edu; Tel.: +1-928-523-0575

**Keywords:** academic identity, quiet ego, self-esteem, cognitive reappraisal, self-handicapping, self-affirmation

## Abstract

The transition to college can be psychologically demanding. This study examines how a more mature and consolidated academic identity (AI) is related to three types of self-regulation strategies in college students during their first semester: cognitive reappraisal (CR), self-handicapping (SH), and self-affirmation (SA). Two self-related resources, quiet ego (QE)—a compassionate, growth-oriented self-identity—and self-esteem (SE)—an individual’s global self-assessment of self-worth—were theorized as complementary, but also unique, predictors of the relationship between AI and self-regulation strategies. QE reflects a less defensive, growth/balance-oriented self-structure with implications for self-regulation and adaptive development above and beyond SE. A multiple regression model testing only indirect effects was tested using R (*lavaan*) in a sample of first-semester college students (N = 352). The hypothesized model fit was acceptable (with five of six indirect hypotheses supported), but a model that added a direct path from AI to SH significantly improved fit without altering the indirect effect results. AI was positively related to CR and SA via both QE and SE, with the path via SE being the strongest. AI was related to lower SH both directly and indirectly only via QE. These results support and add to the literature on the benefits of QE and SE as important yet distinct psychosocial resources for college students. Implications for strengthening QE resources in first-year students are discussed.

## 1. Introduction

The transition to college can be psychologically demanding, often requiring students to manage academic and social challenges. Academic identity (AI) is a strong predictor of how successfully students manage the transition to college, utilize effective coping strategies, and experience academic success (Berzonsky, 1997). AI is the product of two core processes, exploration and commitment (Marcia, 1966; Was & Isaacson, 2008), that combine to create four identity statuses: achieved (high exploration, high commitment), moratorium (high exploration, low commitment), foreclosure (low exploration, high commitment), and diffusion (low exploration, low commitment) (Marcia, 1966; Schwartz et al., 2010).

College students with an achieved AI status have explored their academic options and values (e.g., goals, priorities, persistence, reasons for college, and likely major/career paths) and have committed themselves to an academic direction (Was & Isaacson, 2008). Of the four identity statuses, an achieved AI status has been shown to have the most robust relationship with students’ achievement, well-being, and satisfaction with college (Abdellatif, 2023; Dastjerdi et al., 2022). For example, during the transition to college, a student with an achieved status might meet with an academic advisor early, map how course requirements can be met in their first year, and establish simple routines. If they feel homesick or overwhelmed, they would be more likely to interpret it as normal transition stress rather than a sign that they do not belong. They might say, “This is hard, but I’ve dealt with difficult things before and I know I can do it.” In contrast, less committed and explored AI statuses are likely associated with poorer self-regulation in context, reflected in more avoidant coping and less effective/less reflective decision-making styles (Berzonsky, 1992; Luyckx et al., 2013). Thus, achieved AI is an important psychosocial resource during the college years and can affect the types of coping and self-regulatory strategies adopted in academic settings (Schwartz et al., 2010; Was, 2009). The goal of this study is to examine the extent to which AI is related to three specific types of self-regulatory strategies used to cope with perceived threat: cognitive reappraisal (CR), self-handicapping (SH), and self-affirmation (SA). In the next section, each self-regulation strategy is described.

### 1.1. Self-Regulation Strategies

CR is an emotion-regulation strategy in which a student changes how they interpret a situation in order to change its emotional impact (Gross & John, 2003). For college students, academic stressors are meaningful and often relevant to their academic self-identities (e.g., grades, feedback, and competitive comparisons). CR is especially valuable for students—the ability to reframe threatening information can preserve positive emotion (or at least limit negative emotion). When an academic self-identity is protected, students are more likely to stay engaged and perform effectively. In a field experiment, exposure to a brief reappraisal message about test anxiety improved first-year students’ exam and course performance (Brady et al., 2018). More mature identity statuses (e.g., achieved and moratorium) have been linked with adaptive (e.g., mastery) goal orientations, which posits that stronger perceived control/value promotes more constructive emotional responses and regulation under challenge (Pekrun, 2006; Was, 2009).

SH is a self-protective strategy in which a student creates or claims obstacles (e.g., procrastination, reduced effort, and claiming “I didn’t study”) so that possible failure can be blamed on the obstacle rather than on low ability. To ease performance anxiety, students can procrastinate, distract themselves, or exert lower effort, which then increases the likelihood of poorer outcomes and perpetuates a threat-avoidance cycle that is reliably associated with lower academic achievement (Schwinger et al., 2014). As a defensive reaction to threat, SH may successfully regulate short-term anxiety and protect SE, but ultimately undermines preparation, learning, and performance (Urdan, 2004; Schwinger et al., 2014). AI has been empirically linked to SH among undergraduates, with achieved statuses showing lower SH relative to less mature statuses (Chorba et al., 2012).

Self-affirmation theory argues that people are motivated to maintain a sense of adequacy, and affirming valued aspects of the self can protect SE, reduce defensiveness, and promote more open, adaptive responses to threat (Steele, 1988; Sherman & Cohen, 2006). Self-affirmation strategies, such as reflecting on past achievements and important goals, have been shown to help improve academic outcomes in educational settings (G. L. Cohen et al., 2006; Miyake et al., 2010) by altering students’ psychological experience of threat (G. L. Cohen & Sherman, 2014). In the academic domain, an achieved identity predicts stronger identity affirmation, which is conceptually aligned with the “affirm valued aspects of the self” mechanism outlined in self-affirmation theory (Harris et al., 2019; Pietersma & Dijkstra, 2012). Recent meta-analytic work suggests that SA processes can reduce defensiveness and promote well-being in educational settings (Escobar-Soler et al., 2024; Wu et al., 2021; Zhang et al., 2025). Taken together, SA is likely a strategy associated with a more mature AI (Steele, 1988; G. L. Cohen & Sherman, 2014).

### 1.2. Self-Related Processes

The self is an organized and dynamic system supported by several psychosocial mechanisms. This study focuses on two, quiet ego (QE) and self-esteem (SE), which are distinct but complementary resources that can help explain the relationship between AI and self-regulation strategies. QE describes an *identity-structure*, a pattern of self-organization whereby the self is not erased but is integrated with awareness of others, openness to growth, and less egoic reactivity (Bauer & Weatherbie, 2023). SE describes an *evaluative stance* regarding the self—whether one sees themselves positively or negatively—and is often associated with self-enhancing tendencies (Taylor et al., 2003). Both QE and SE support well-being, but through somewhat different routes. QE reflects, “How do I hold and integrate my concern for self with concern for others?”, whereas SE reflects, “How good do I feel about myself?” In the paragraphs that follow, QE and SE are described, including how they are related to self-regulatory strategies.

QE is a mid-level self-identity construct characterized by four inter-related characteristics of detached awareness, inclusive identity, perspective taking, and growth orientation (Bauer & Weatherbie, 2023; Wayment et al., 2015). QE—a compassionate, less defensive way of construing the self in relation to others—has been identified as a positive indicator of flourishing and life satisfaction across samples (Pradhan et al., 2025; Rogawski et al., 2025) and lower stress among college students mediated via self-control, self-compassion, and pursuing compassionate goals (Wayment et al., 2016; Wayment & Cavolo, 2018; Wayment & Bauer, 2017; Wayment et al., 2019).

The connection between QE and AI has not yet been explored; however, there are reasons to expect such a relationship. In the context of confronting academic stressors, more mature forms of AI are commonly associated with more open and self-reflective identity processing, and less avoidant coping and more deliberate, problem-focused coping. For example, students with more mature AIs can hold academic commitments with greater clarity and flexibility (rather than threat reactivity), which should make it easier to stay growth-focused, be open to new perspectives, and maintain self–other balance during setbacks—core features of QE (Wayment et al., 2015; Wayment & Bauer, 2017). Given that QE is a self-identity that supports non-defensive self-awareness and constructive self-criticism (Kesebir, 2014), QE should be positively related to self-affirming strategies because SA reduces defensiveness by affirming important values/strengths when threatened (Steele, 1988; G. L. Cohen & Sherman, 2014), including focusing on important social relationships (Crocker et al., 2008). QE should also be negatively related to SH because SH is a classic defensive self-protection strategy, whereas QE is explicitly characterized by *lower defensiveness* and a more secure, growth-oriented response to threat (Wayment & Bauer, 2017; Kesebir, 2014). Finally, QE should be positively related to self-regulatory efforts, including CR and SA. The QE components of detached awareness and growth-mindedness are conceptually compatible with more adaptive emotional regulation strategies (Gross & John, 2003; Cutuli, 2014). In previous research, QE has been positively associated with CR (Wayment & Cavolo, 2018; Wayment et al., 2019). Regarding SA, a relationship with QE is expected, but the size of the relationship is expected to be modest. QE is not the elimination of self-concern, but a *balance* of self- and other-concern. Critcher and Dunning (2015) review the literature on SA and make the argument that SA puts the “self-in-perspective” which aligns SA with fundamental features of QE. A recent experimental study found support for this perspective, with reminders of QE ideas associated with reduced self–other bias (Wayment, 2025).

SE reflects a student’s global sense of self-worth (Rosenberg, 1965) and is robustly associated with subjective well-being. Academic and social challenges can repeatedly test students’ sense of competence during the transition to college. When SE is secure, students are less likely to interpret ordinary academic struggles as evidence of personal inadequacy and are more likely to persist, seek support, and rebound after any setbacks. More mature forms of AI should relate positively to SE because an achieved identity is typically associated with effective goal orientation and academic achievement (Hejazi et al., 2012; Marcia, 1966; Orlofsky, 1978; Ryeng et al., 2013). More mature AI statuses have been shown to relate to students’ motivational orientations in ways that reinforce key features of SE, perceived competence, and self-worth (Was, 2009). A meta-analysis of identity-status studies found that individuals with more mature statuses reported higher SE than those with less mature statuses (Ryeng et al., 2013).

SE is related to self-regulation strategies because such strategies are helpful in managing threats to self-worth and self-integrity (Tangney et al., 2004). As discussed earlier, SA (reflecting on core values or important strengths) is widely discussed as a way to protect SE; thus, SE is expected to be positively related to SA (Steele, 1988; Sherman & Cohen, 2006; G. L. Cohen & Sherman, 2014). Some forms of high SE are believed to be more fragile (e.g., contingent) and accompanied by defensiveness (Crocker & Park, 2004; Kernis et al., 2008). Thus, it could be that since SH is often used as a SE-protective strategy in performance contexts, students preserve self-worth by providing an external explanation for possible failure (Berglas & Jones, 1978). Thus, it is expected that SE will be positively related to SH, setting up a competing hypothesis with QE (see H2a and H2b below). Finally, SE has been found to be positively related to CR (Gross & John, 2003) and is expected in this study as well.

Taken together, QE and SE are hypothesized to serve as explanatory mechanisms for the relationship between AI and three self-regulatory strategies. Indeed, although both SE and QE have been shown to be modestly correlated and each related to some specific self-regulation processes, their potentially independent roles in predicting specific self-regulation strategies in college students have not been examined.

**Figure 1:**
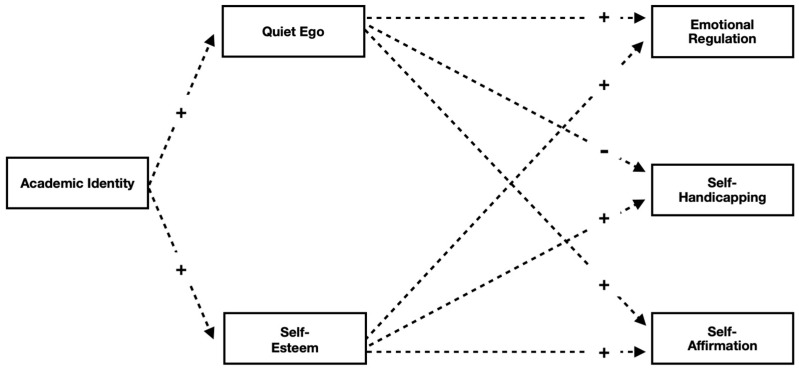
Hypothesized indirect effects model. + indicates positive relationship; − indicates negative relationship. Dashed lines represent paths used to calculate indirect effects.

An indirect effects model (see Figure 1) was tested in a sample of college students during their first semester of college.

**H1.**  *AI will be positively associated with CR via both QE and SE.*

**H2a.**  *AI will be negatively associated with SH via QE.*

**H2b.**  *AI will be positively associated with SH via SE.*

**H3.**  *AI will be positively related to SA via QE and SE.*

Although not pictured in the figure, several correlational relationships are also expected. QE and SE will be modestly correlated with one another, SA is expected to be modestly and positively associated with CR, and SA is expected to be negatively related to SH.

## 2. Materials and Methods

### 2.1. Participants and Procedures

Participants were first-semester PSY 101 students (N = 352) recruited through the department participant pool (SONA) for course credit during the 2024 fall semester. All study procedures were approved by the institution’s IRB (#2221512). Participants completed a survey online delivered by Qualtrics. The sample consisted primarily of female (N = 291, 82.7%) and male (N = 45, 12.8%) students, with 5 identifying as non-binary (N = 5, 1.4%). The average age was 18.54 (SD = 1.25). The ethnicity breakdown was as follows: White (N = 186, 52.8%), Hispanic (N = 60, 17%), Hispanic/White (N = 32, 9.1%), Asian (N = 13, 3.7%), and AI/AN (N = 6, 1.7%). Ten respondents did not answer, and the remaining respondents indicated a combination of the primary ethnic categories (N = 45, 12.8%).

### 2.2. Measures

AI was measured using a subset of 12 items measuring academic goals selected from the original 40-item Academic Identity Measure (AIM; Was & Isaacson, 2008).^1^ Participants responded to each of the items on a 5-point scale (1 = *not at all like me*; 5 = *very much like me*). The items were subjected to an exploratory factor analysis, revealing strong support for a single factor.^2^ Next, the 12 items were averaged to create a measure of AI with a coefficient alpha of 0.78. The measure is best conceptualized as a unidimensional construct reflecting higher endorsement of three achieved identity items, lower endorsement of three diffusion and three moratorium items, and little to no endorsement of three foreclosure items. Higher scores on this measure reflect a more mature, consolidated, and committed sense of AI.

QE was assessed with the full 14-item Quiet Ego Scale (QES) (Wayment et al., 2015). The items captured four facets (detached awareness, inclusive identity, perspective taking, and growth-mindedness) and were rated on a 5-point scale (1 = *strongly disagree*; 5 = *strongly agree*). Sample items included “I feel a connection with all living things,” “I am quick to learn from my mistakes,” and reverse-keyed items such as “I find myself doing things without paying much attention.” In their meta-analytic review (41 studies), Rogawski et al. (2025) reported that the reliability estimates ranged from 0.68 to 0.91 (median α = 0.77). Coefficient alpha in this sample was 0.72.

SE was assessed with a five-item short form of the Rosenberg Self-Esteem Scale (RSE; Rosenberg, 1965) using a 5-point scale (1 = *strongly disagree*; 5 = *strongly agree*). Sample items included: “On the whole, I am satisfied with myself,” “At times I think I am no good at all” (R), and “I take a positive attitude toward myself.” Reverse-keyed items were recoded, and the items were averaged such that higher values indicated higher SE. Coefficient alpha in this sample was 0.85.

CR was assessed using the cognitive reappraisal subscale from the Emotion Regulation Questionnaire (ERQ; Gross & John, 2003). Five items were rated using a 5-point scale (1 = *strongly disagree*; 5 = *strongly agree*), and none required reverse scoring. One item (“When I want to feel less negative emotion, I change the way I’m thinking about the situation I’m in”) was inadvertently omitted from the survey. A representative ERQ reappraisal item was “When I want to feel more positive emotion, I change what I’m thinking about.” Scores were averaged so that higher values indicated more frequent reappraisal. Coefficient alpha in this sample was 0.81.

SH was assessed using five items drawn from the five highest loading defensive items in the Self-Enhancement and Self-Protection Strategies measure (Hepper et al., 2011). These items reflect behaviors and cognitions that protect the self under threat (including SH and discounting), such as “Reviewing very little for a test…so that if you do poorly, it would not mean you are incompetent” and “When you do poorly at something, thinking the situation or test was uninformative/inaccurate” (Hepper et al., 2011). Responses were made on a sliding 6-point scale (1 = *not at all characteristic of me*; 6 = *very characteristic of me*). Hepper et al. (2011) reported strong reliability for the Defensiveness factor (0.83 to 0.86). Coefficient alpha in the sample was 0.71.

SA was measured with the six-item Cognitive Self-Affirmation Inclination (CSAI) scale (Pietersma & Dijkstra, 2012), which assesses people’s tendency to spontaneously access positive self-images when threatened. Items were rated on a 5-point scale (1 = *never*; 5 = *very often*) and included content such as noticing one’s strengths (e.g., “I notice I do some things very well”). Items were averaged so that higher scores reflected greater SA inclination. The CSAI scale demonstrated good internal consistency (0.81; Pietersma & Dijkstra, 2012). Coefficient alpha in the sample was 0.79.

## 3. Results

### 3.1. Analytic Strategy

All analyses were conducted using *R* (v. 4.4.2). Pairwise deletion methods were used to compute descriptive information. For the correlation matrix, listwise deletion methods were used. Full information maximum likelihood (FIML) was used to estimate the model using all available data, reducing bias compared to listwise deletion. Study variables had adequate skewness (range: −0.60 to 0.45) and kurtosis (−0.70 to 0.03) estimates.

### 3.2. Descriptive Results

Summary statistics for the study variables are listed in Table 1.

Correlations among the study variables were computed and are presented in Table 2. The correlations largely confirmed the expectations regarding hypothesized relationships that would ultimately be tested in a multiple regression model. AI was positively related to both QE and SE. QE was positively correlated with all three strategies in the expected directions. QE was also correlated with CR and SA in expected directions. Contrary to expectation, SE was negatively (not positively) related to SH. Among the three self-regulation strategies, the only significant relationship was between SA and CR.

### 3.3. Multiple Regression Results

**Figure 2:**
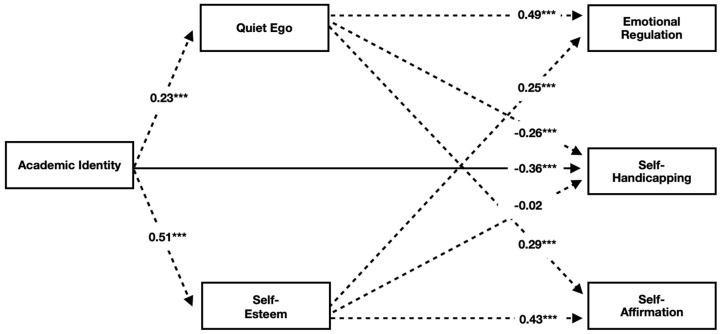
Final model results. The solid line represents the direct path that was added to the hypothesized model; dashed lines are the initial, hypothesized, indirect paths. *** *p* < 0.001.

**Figure A1:**
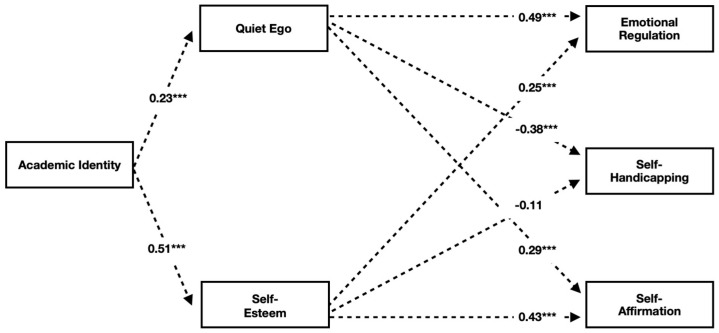
Hypothesized indirect effects model results. *** *p* < 0.001.

Given the expected shared variance among study variables, the hypothesized indirect effects model (see Figure 1) was tested with a multiple regression model in R (*lavaan*) to estimate the hypothesized predictor’s unique contribution to the outcome variables while controlling for the other predictors (J. Cohen et al., 2003). See Table A1 and Figure A1 in Appendix A for the full results, including fit indices and all path coefficients, including 90% CIs. Support was found for five of the six hypothesized indirect effects. Although the hypothesized model had a relatively acceptable fit (X^2^_(5)_ = 17.713, *p* < 0.003, CFI = 0.97, RMSEA = 0.085, SRMR = 0.039), post hoc information suggested that adding one path (AI to SH) to the model would increase model fit. The updated model was retested (X^2^_(4)_ = 1.779, *p* = 0.776, CFI = 1.00, RMSEA = 0.00, SRMR = 0.00). The added direct path from AI to SH improved model fit (X^2^_(1)_ = 15.934, *p* < 0.0001) without changing the pattern of indirect effects results from the hypothesized model. The model results are depicted in Figure 2. AI was indirectly related to all three self-regulation strategies via QE (H1, H2a, and H3). Two of three hypotheses for SE were supported (H1 and H3), showing that AI was indirectly related to CR and AI via SE. However, given that SE was not related to SH, there was no significant indirect effect from AI to SH via SE (H2b).

## 4. Discussion

This study integrates QE into the achievement identity framework by examining the unique and shared contributions of QE and SE to self-regulation strategies in first-semester college students. The model also assumed that QE and SE would both be positively associated with a more mature AI. QE reflects an integrated, growth-oriented self that balances self-concern with concern for others and responds to threat with less defensiveness (Bauer & Weatherbie, 2023; Rogawski et al., 2025; Wayment et al., 2015). A more mature and consolidated AI should also be positively associated with SE, understood as a stable, secure, global, and noncontingent positive self-regard and sense of self-worth (Crocker & Park, 2004; Rosenberg, 1965). In the original QES validation work, Wayment et al. (2015) reported that QES was meaningfully associated with several outcomes even when controlling for SE, suggesting that they have potentially overlapping and unique roles in the self-system and that QE reflects a broader, more self–other-integrative self-structure than SE-like indicators alone. Accordingly, the research described here sought to examine the shared and unique effects of QE and SE on specific self-regulation strategies.

The results also show the complementary nature of QE and SE. In this study, AI was associated both with positive self-regard (SE) and a compassionate, growth-focused view of self and others (QE). The regression results revealed that SE captured a much larger portion of the variance in AI than did QE, consistent with prior research showing a strong relationship between achieved forms of identity with SE (Ryeng et al., 2013). However, QE was also an important explanatory variable, suggesting QE and SE are complementary yet unique resources associated with AI. Prior research on the QE construct has suggested that QE captures a broader growth-oriented self-and-other system beyond SE alone (Bernabei et al., 2024).

All three types of self-regulation strategies examined are self-protective strategies—a way to maintain self-integrity in the face of threat. Of the three, only SH is seen as a “defensive” strategy, whereas SA and CR are methods that help students remain open-minded, flexible, and adaptive in the face of threat. These two strategies help students navigate threats to their self-worth or emotional state by shifting their focus to positive emotion, past successes, and important values. Accordingly, these two strategies were, as expected, moderately correlated, and SH, the more defensive strategy, was unrelated to both CR and SA. The zero-order correlations showed that AI was most strongly and positively associated with SA and negatively associated with SH. There was also a modest positive correlation with CR.

The model tested in this study was designed to examine how QE and SE could help explain the relationship between AI and these three strategies. AI was indirectly related to SA most strongly via SE. This result is very consistent with the literature on SA and its role in protecting SE (Steele, 1988; G. L. Cohen & Sherman, 2014). The indirect effect with QE was also significant, but to a much lesser degree, and is consistent with research showing that when QE characteristics are salient, the types of affirmations made tend to be more other-focused (Wayment, 2025) and may not be adequately captured by items on the SA measure used in this study.

Both QE and SE helped explain the relationship between AI and CR; the indirect effects were nearly equivalent, but modest. Given the shared variance between SA and CR, it is unclear what the unique variance in CR (after controlling for SA) captured. Perhaps it was the ability to shift one’s thinking when needed, or reflected the goal of maintaining a positive mood. More research is needed to fully understand this result.

Finally, only QE, and not SE, helped to explain the negative indirect effect from AI to SH. QE was uniquely associated with lower defensiveness, consistent with the idea that QE reflects a less self-protective and more growth-oriented self-identity (Bauer & Weatherbie, 2023; Wayment et al., 2015). The lack of a relationship with SE could be due to several causes. Motives for SH may differ depending on whether SE is secure or unstable and whether motives are to protect or enhance SE (Crocker & Park, 2004; Tice, 1991). Our results with SE in the overall model support earlier research showing that SE is typically linked to more adaptive regulation patterns (e.g., greater use of CR and less reliance on suppression), which support better emotional functioning and interpersonal outcomes (Gross & John, 2003).

The hypothesized model fit was improved by adding a direct effect from AI to SH, suggesting a primary role of a more mature AI in refraining from greater use of this defensive strategy. This result is consistent with research showing that college students with more mature AIs (e.g., achieved) are not motivated by anxiety or fear of failure, affective states that prompt SH behavior (Chorba et al., 2012; Hejazi et al., 2012). Students with more consolidated AIs have a mastery-oriented focus and see themselves as capable and motivated by achievement, and experience less uncertainty and anxiety regarding their abilities (Was & Isaacson, 2008).

Taken together, the present findings extend existing research in several ways. They suggest that a more mature academic self-identity is not only relevant for performance and retention, but also for how students relate to themselves—whether they adopt a confident and compassionate stance toward themselves and others, how defensive they become, and how readily they affirm what they value about themselves. These findings show that an academic self-identity can function as an upstream resource that shapes students’ self-regulation strategies through its effects on the two important self-related resources used in this study: QE and SE. Results demonstrate that students with more consolidated and mature academic identities tend to report greater self-worth and a more compassionate and growth-oriented stance toward the self and others, which is associated with healthier self-regulation strategies.

Key limitations of this research include the cross-sectional design, self-report measurement of general tendencies, sample size, and the use of a college student sample. Although the results are in line with expectations, they are modest. The cross-sectional design rules out causality and only describes associations between self-report measures. Thus, we cannot infer causal relationships between AI, QE, SE, and self-regulation strategies. An important limitation concerns the measurement of AI. In the present study, the use of a subset of AIS items led to a unidimensional measure reflecting more consolidation and commitment to one’s academic goals, but not the four discrete statuses as outlined by Marcia (1966). Future studies on this topic would benefit from using the full AIS to be able to examine the four identity statuses separately.

The results presented here are not generalizable to other college populations or adults in general (Henrich et al., 2010). The very high proportion of female respondents is also problematic and precluded any analysis of potential sex differences. The cross-sectional study design with a moderately sized sample requires that the results be interpreted as preliminary and with caution. Although the sample size provided adequate sensitivity for the magnitude of results found in this study, null findings may reflect limited precision for small effects and should be interpreted in light of their confidence intervals. Experimental studies manipulating types of threatening academic feedback and the types of strategies students use in real-time would be extremely informative. Experimental work could also test whether brief interventions that target academic self-identity, QE, or SE could also impact students’ self-regulatory choices.

Many types of services are used to support students transitioning to college, including peer-mentoring, first-year seminars, or advising (Padgett & Keup, 2011). Findings from this study have some practical implications for such programming. Specifically, QE can be strengthened through brief interventions (see Liu et al., 2022; Wayment, 2025; Wayment et al., 2019 for examples) and could be integrated into popular sense of belonging interventions (Le et al., 2024) that focus on reminding students that adversities are normal and short-lived (Davis et al., 2019; Murphy et al., 2020). Incorporating QE content could provide students with specific strategies to reframe ambiguous or threatening experiences as a part of normal adaptation to college. For example, detached awareness can reduce self-serving biases; perspective taking can help students interpret others’ actions more charitably; inclusive identity helps students respond with empathy and shared humanity; and growth helps reframe uncertainty and mistakes as a normal part of growth. Incorporating QE content into self-esteem interventions also aligns with suggestions that realistic self-appraisal and self-compassion can help strengthen less fragile forms of SE (Baumeister et al., 2003; Crocker & Park, 2004; Neff & Vonk, 2009). Institutional advising efforts might help students articulate *why* they are in college, what kind of student they want to be, and how their studies connect to their *valued life goals*. In first-year seminars, structured reflection and narrative-writing activities could help students think more about their AI (Lilgendahl & McLean, 2020). In this context, discussions of how important self-processes (QE and SE) lead to productive and unproductive self-regulation strategies could be helpful for students.

Future studies might also examine for whom self-identity supporting interventions would be most helpful. Self-regulatory processes have been identified as particularly important for first-generation students, students from underrepresented groups, and those experiencing higher academic or financial stress. In addition, replicating this work in other cultural contexts would help determine the generalizability of the findings beyond a single sample (cf., Bernabei et al., 2024; Davis et al., 2019; Le et al., 2024; Murphy et al., 2020).

## Figures and Tables

**Table 1:** Study variables (N = 352).

Measure	N	M	SD
Academic Identity	352	3.74	0.61
Quiet Ego	352	3.58	0.43
Self-Esteem	350	3.31	0.78
Cognitive Reappraisal	348	3.49	0.70
Self-Handicapping	340	2.59	0.93
Self-Affirmation	352	2.90	0.69

**Table 2:** Correlations among study variables (complete cases N = 336).

Variable	AI	QE	SE	CR	SH
Academic ID (AI)	---				
Quiet Ego (QE)	0.322 ***	---			
Self-Esteem (SE)	0.379 ***	0.290 ***	---		
Cognitive Reappraisal (CR)	0.149 **	0.374 ***	0.357 ***	---	
Self-Handicapping (SH)	−0.285 **	−0.204 **	−0.147 **	−0.078	---
Self-Affirmation (SA)	0.237 ***	0.335 ***	0.554 ***	0.438 ***	−0.076

*Note*: ** *p* < 0.01; *** *p* < 0.001.

## Data Availability

The original data presented in the study are openly available in OSF at https://osf.io/jhzxd/overview?view_only=4536cee51e9845c7b5995b4e68391a50, accessed on 20 February 2026.

## Notes

1 The three highest loading items reported for each identity status were used (cf., Was & Isaacson, 2008). Participants responded to each of the items 195 on a 5-point scale (1 = not at all like me; 5 = very much like me). The three achieved identity 196 items were “Although I have many priorities, learning in school is always one of my most 197 important goals,” “A college education is a high priority for me and I’m willing to make the sacrifices,” and “I know why I am in college and have clear goals I want to achieve.” The remaining nine items reflected the identity statuses that were low in exploration and/or commitment (i.e., less mature statuses) and included “I don’t have clear priorities for school and life. I usually just go with the flow” (diffused identity), “My priorities in school are in transition. Some days I am serious, other days I have other priorities” (moratorium identity), and “When I do poorly on a test I get upset and worry what friends and family might think of me” (foreclosed identity).

2 Moratorium, diffused, and foreclosed items were reversed. Eigenvalue for a single factor solution was 3.35, capturing 28% of the variance across the 12 items.

## Abbreviations

The following abbreviations are used in this manuscript:

AI	Academic Identity
QE	Quiet Ego
SE	Self-Esteem
CR	Cognitive Reappraisal
SH	Self-Handicapping
SA	Self-Affirmation

## Appendix A

**Table A1:** Summary of model results (N = 352, 4 incomplete cases).

		Hypothesized Model	Final Model
Fit Indices	Parameters	20	21
	Chi-Square (X^2^)	17.713	1.779
	df	5	4
	*p*	0.003	0.776
	X^2^/df ^†^	3.54	0.445
	CFI	0.966	1.00
	TLI	0.899	1.02
	RMSEA	0.085	0.000
	[90% CI]	[0.044, 0.133]	[0.000, 0.053]
	SRMR	0.039	0.000
Paths	AI → QE	0.227 [0.160, 0.294]	0.227 [0.160, 0.294]
	AI → SE	0.514 [0.390, 0.639]	0.514 [0.390, 0.639]
	QE → ER	0.487 [0.327, 0.647]	0.487 [0.327, 0.647]
	SE → ER	0.249 [0.150, 0.349]	0.249 [0.150, 0.349]
	QE → SH	−0.375 [−0.621, −0.130]	−0.256 [−0.500, −0.012]
	SE → SH	−0.110 [−0.260, 0.039]	−0.022 [−0.187, 0.144]
	QE → SA	0.291 [0.146, 0.436]	0.291 [0.146, 0.436]
	SE → SA	0.433 [0.361, 0.505]	0.433 [0.361, 0.505]
	AI → SH	---	−0.364 [−0.557, −0.171]
Indirect Effects	AI to ER via QE	0.111 [0.062, 0.160]	0.111 [0.064, 0.160]
	AI to ER via SE	0.128 [0.074, 0.183]	0.128 [0.074, 0.183]
	AI to SH via QE	−0.085 [−0.148, −0.023]	−0.058 [−0.114, −0.002]
	AI to SH via SE	−0.057 [−0.135, 0.021]	−0.011 [−0.096, 0.074]
	AI to SA via QE	0.066 [0.029, 0.103]	0.066 [0.029, 0.103]
	AI to SA via SE	0.223 [0.157, 0.289]	0.223 [0.157, 0.289]

^†^ X^2^/df normalizes the chi-square statistic by the model’s degrees of freedom to assess fit, with lower values indicating better fit, generally aiming for ≤3 or 5, though interpretations vary, with some suggesting <2 for an excellent fit. This index helps adjust for model complexity, as the raw chi-square test is highly sensitive to sample size, often flagging large models as poor fits even when good.
